# Hypothalamus and intracranial volume segmentation at the group level by use of a Gradio-CNN framework

**DOI:** 10.1007/s11548-025-03438-6

**Published:** 2025-06-06

**Authors:** Ina Vernikouskaya, Volker Rasche, Jan Kassubek, Hans-Peter Müller

**Affiliations:** 1 Department of Internal Medicine II, Ulm University Medical Center, Albert-Einstein-Allee 23, 89081 Ulm, Germany; 2 Department of Neurology, University of Ulm, Ulm, Germany

**Keywords:** MRI, Hypothalamus, ICV, Segmentation, GUI, CNN, Gradio

## Abstract

**Purpose:**

This study aimed to develop and evaluate a graphical user interface (GUI) for the automated segmentation of the hypothalamus and intracranial volume (ICV) in brain MRI scans. The interface was designed to facilitate efficient and accurate segmentation for research applications, with a focus on accessibility and ease of use for end-users.

**Methods:**

We developed a web-based GUI using the Gradio library integrating deep learning-based segmentation models trained on annotated brain MRI scans. The model utilizes a U-Net architecture to delineate the hypothalamus and ICV. The GUI allows users to upload high-resolution MRI scans, visualize the segmentation results, calculate hypothalamic volume and ICV, and manually correct individual segmentation results. To ensure widespread accessibility, we deployed the interface using ngrok, allowing users to access the tool via a shared link. As an example for the universality of the approach, the tool was applied to a group of 90 patients with Parkinson’s disease (PD) and 39 controls.

**Results:**

The GUI demonstrated high usability and efficiency in segmenting the hypothalamus and the ICV, with no significant difference in normalized hypothalamic volume observed between PD patients and controls, consistent with previously published findings. The average processing time per patient volume was 18 s for the hypothalamus and 44 s for the ICV segmentation on a 6 GB NVidia GeForce GTX 1060 GPU. The ngrok-based deployment allowed for seamless access across different devices and operating systems, with an average connection time of less than 5 s.

**Conclusion:**

The developed GUI provides a powerful and accessible tool for applications in neuroimaging. The combination of the intuitive interface, accurate deep learning-based segmentation, and easy deployment via ngrok addresses the need for user-friendly tools in brain MRI analysis. This approach has the potential to streamline workflows in neuroimaging research.

## Introduction

The accurate segmentation of the hypothalamus has recently gained increasing attention in neuroscience research. As a multi-integral brain region, the hypothalamus regulates critical physiological processes, including hormonal balance, thermoregulation, sleep, and emotional responses [1]. Precise delineation of the hypothalamus in magnetic resonance imaging (MRI) data enables deeper insights into its structural changes associated with neurological diseases like, e.g., Alzheimer’s disease [2], Parkinson’s disease (PD) [3], amyotrophic lateral sclerosis (ALS) [4], Huntington’s disease [5] or metabolism-associated disorders like idiopathic intracranial hypertension [6]. However, manual segmentation of the hypothalamus is time-consuming, labor-intensive, and prone to variability, requiring trained professionals to achieve consistent results. To address these challenges, there has been a growing emphasis on developing automatic segmentation methods that can provide accurate, reproducible, and efficient analysis, thereby enhancing both research outcomes and clinical applications [7, 8].

In recent years, convolutional neural networks (CNNs) have demonstrated remarkable performance in medical image segmentation, including applications involving complex brain structures like the hypothalamus [9–12]. CNN-based approaches are able to learn hierarchical features representing different levels of abstraction in a data-driven manner [13], enabling them to accurately segment different structures with high precision. These automated methods offer a viable solution for the large-scale analysis of the hypothalamus structure across extensive patient datasets, facilitating population-level studies [7, 14]. However, for these CNN-based segmentation models to be adopted widely, they must be accessible to the public, defining the need for a user-friendly interface that simplifies the process, enabling non-specialists to leverage these powerful tools seamlessly.

To bridge this gap, the use of intuitive graphical user interfaces (GUIs) becomes essential. A GUI allows users to interact with complex segmentation models without needing to understand the underlying code, making advanced imaging technology more approachable and practical for researchers. The Gradio library, an open-source Python package for creating customizable UI components for machine learning models, provides a powerful platform for developing such GUIs. Designed for ease of use, Gradio enables developers to quickly create interactive web-based applications that can run CNN segmentation models with minimal configuration.

The current work builds upon our previous research on AI-assisted quantification of hypothalamic atrophy by CNN-based automatic segmentation to segment both hypothalamus and intracranial volume (ICV) for hypothalamic volume normalization [15], previously applied to ALS as a disease known to be associated with hypothalamic volume reduction [16]. This work presents three key contributions: (1) an improved segmentation methodology that maintains high performance, as evidenced by a comparative study with our previous work; (2) the introduction of a GUI powered by Gradio and ngrok tunneling, offering a comprehensive end-to-end solution from model training to user interaction, accessible through any platform without installation or compatibility concerns; and (3) a proof-of-concept validation case series that demonstrates the practical applicability of our approach.

## Methods

### MRI data and preprocessing

T1-weighted whole head MRI images acquired on a 1.5 T MRI scanner (Symphony, Siemens Medical, Erlangen, Germany) as part of a standard clinical MRI protocol were sourced from the MRI database of the Department of Neurology, University of Ulm, Germany. For this study, we utilized the same dataset as in the previous study [15] which includes 108 MRI volumes from 66 patients with ALS (mean age 61 ± 9 years, 58% male) and 42 healthy controls (mean age 53 ± 17 years, 50% male) without any neurological/psychiatric disease or other medical condition. These 108 MRI volumes were used for training and validation of the hypothalamus segmentation pipeline. For the ICV segmentation, we used 10 MRI volumes from the previous study for training [15].

The original T1-weighted MRI scans were acquired in sagittal orientation with an in-plane resolution of 1.0 mm and a slice thickness of 1.0–1.2 mm, depending on acquisition parameters. As part of the previous study, the images were reconstructed into 3D volumes with an isotropic resolution of 1.0 mm and reoriented to coronal orientation following rigid brain normalization using the *Tensor Imaging and Fiber Tracking (TIFT)* software [17]. During that process, the data were upsampled to higher resolutions, and manual annotations for both hypothalamus and ICV were generated based on these preprocessed images following the well-established manual delineation procedure, which has been used in the previous studies [4], demonstrating a high level of reproducibility. In contrast to our prior study, which employed a data augmentation pipeline involving generation of varying contrast images, the current approach applies a standardized preprocessing step using z-score intensity normalization—based on the mean and standard deviation of the means across all subjects—to reduce intensity variations across images while maintaining the original dataset size. The processed DICOM images were then reformatted into two sets of 2D slices stored in PNG format for training the segmentation networks:

1. Hypothalamus segmentation—each subject’s MRI was stored as 50 pre-selected slices at a resolution of 0.125 × 0.125 × 0.5 mm^3^ (matrix: 512 × 512 pixels).
2. ICV segmentation—full 3D volumes were stored as 512 slices per subject at a resolution of 0.5 × 0.5 × 0.5 mm^3^ (matrix: 512 × 512 pixels).

### Dataset splits and models’ training

For hypothalamus segmentation, the available MRI volumes were subdivided into training and test sets, with a gender- and aged-matched test group of 30 subjects, including 15 ALS patients and 15 controls, consistent with the previous study’s methodology [15] to allow for the direct comparison of the models. In contrast to the previous study, we adopted a five-fold cross-validation strategy, where 20% of the data was held out at each fold as a validation set, for model evaluation instead of using a single model, thus ensuring a more robust performance assessment by the mitigation of potential biases from specific data splits. This approach maximizes the training data available to the model, enhancing its ability to learn complex features and improve generalization. By preserving a fixed test set, we ensure that model performance is evaluated consistently across each fold, providing a reliable assessment of its robustness and reducing variability in test results. This balance between training and testing data enables an effective trade-off, allowing the model to fully leverage the available data while maintaining an unbiased evaluation on a separate test set.

For training, we used a U-Net model with EfficientNetB0 backbone pre-trained on The ImageNet dataset. The training was performed along 25 epochs using early stopping where the training was stopped when the validation loss was observed to have ceased improving for 10 consecutive epochs with a batch size of 4 images per pass on a Nvidia GeForce GTX 1060 6 GB GPU. The loss function was based on the sum of the categorical Cross Entropy and Jaccard loss and Adaptive Moment Estimation (Adam) with the learning rate of 10^–4^ and remaining hyperparameters kept with their default Keras values was used as the optimizer. Mean Intersection over Union (IoU) was used as metric to evaluate the model.

For inference, the weighted ensemble of the five models obtained from five-fold cross-validation, was used for hypothalamus segmentation, with fixed weights derived from the normalized validation IoU scores at each fold. Additionally, each individual model from five folds was independently evaluated on the test set, which included both ALS patients and healthy controls, to provide a comprehensive understanding of model variability and generalization across different training subsets and different clinical conditions.

Given the robustness of the most frequently used open-source automated ICV estimation tools, such as FreeSurfer (eTIV/sbTIV)^1^ [18, 19], SPM12^2^ [19, 20], CAT12^3^ [19], or FSL^4^ [19], the segmentation of ICV from MRI images can be considered as a relatively straightforward and well-defined task for CNN-based approaches due to excellent contrast between brain tissue, cerebrospinal fluid, and surrounding structures. Moreover, ICV is a clearly defined anatomical region with consistent boundaries across individuals, which simplifies the segmentation process. Therefore, since only 10 MRI volumes (512 slices each) were available with the corresponding ground truth for training of the ICV model, we employed a single-validation scheme for training, leaving one case as a validation set. All other training parameters were kept consistent with those used for the hypothalamus model. Additionally, we utilized four MRI volumes that were already part of the hypothalamus test dataset and manually generated corresponding ICV annotations for them using identical delineation protocol as this applied in the training data using visual intensity-based three-dimensional marking tool within the *TIFT* software. This independent test set included scans from two healthy control subjects and two ALS patients, enabling quantitative and qualitative evaluation of the models’ generalizability across both subject groups.

### Performance estimation

Performance metrics, including Dice score, 95th quantile Hausdorff distance, and volume similarity [21], were assessed to evaluate the segmentation accuracy of the hypothalamus and ICV in the test datasets. Dice score measures the overlap between the predicted and ground truth segmentation and is defined as:

$$ {\text{Dice}} = \frac{{2\left| {A \cap B} \right|}}{\left| A \right| + \left| B \right|} = \frac{{2{\text{TP}}}}{{2{\text{TP}} + {\text{FP}} + {\text{FN}}}}, $$

where $A$ is the predicted segmentation and $B$ is the ground truth, and $\text{TP}$, $\text{FP}$, and $\text{FN}$ denote true positives, false positives, and false negatives.

To assess boundary accuracy, we included the 95th quantile Hausdorff distance (${\text{HD}}_{95}$) which mitigates sensitivity to extreme outliers and is defined as:

$$ {\text{HD}}_{95} \left( {A, B} \right) = \max \left\{ {h_{95} \left( {A,B} \right),h_{95} \left( {B,A} \right)} \right\}, $$

where ${h}_{95}\left(A,B\right)$ is the 95th percentile of the minimum Euclidean distance between points in $A$ and the closest points in $B$.

Additionally, volume similarity ($\text{VS}$) was calculated to assess the consistency of volumetric measurements, using the formula:

$$ {\text{VS}} = 1 - \frac{{\left| {\left| A \right| - \left| B \right|} \right|}}{\left| A \right| + \left| B \right|} = 1 - \frac{{\left| {{\text{FN}} - {\text{FP}}} \right|}}{{2{\text{TP}} + {\text{FP}} + {\text{FN}}}} $$

These metrics are reported as the mean and standard deviation across cross-validation folds, including results for each individual model and the averaged ensemble of all five models.

### Segmentation GUI

**Fig. 1:**
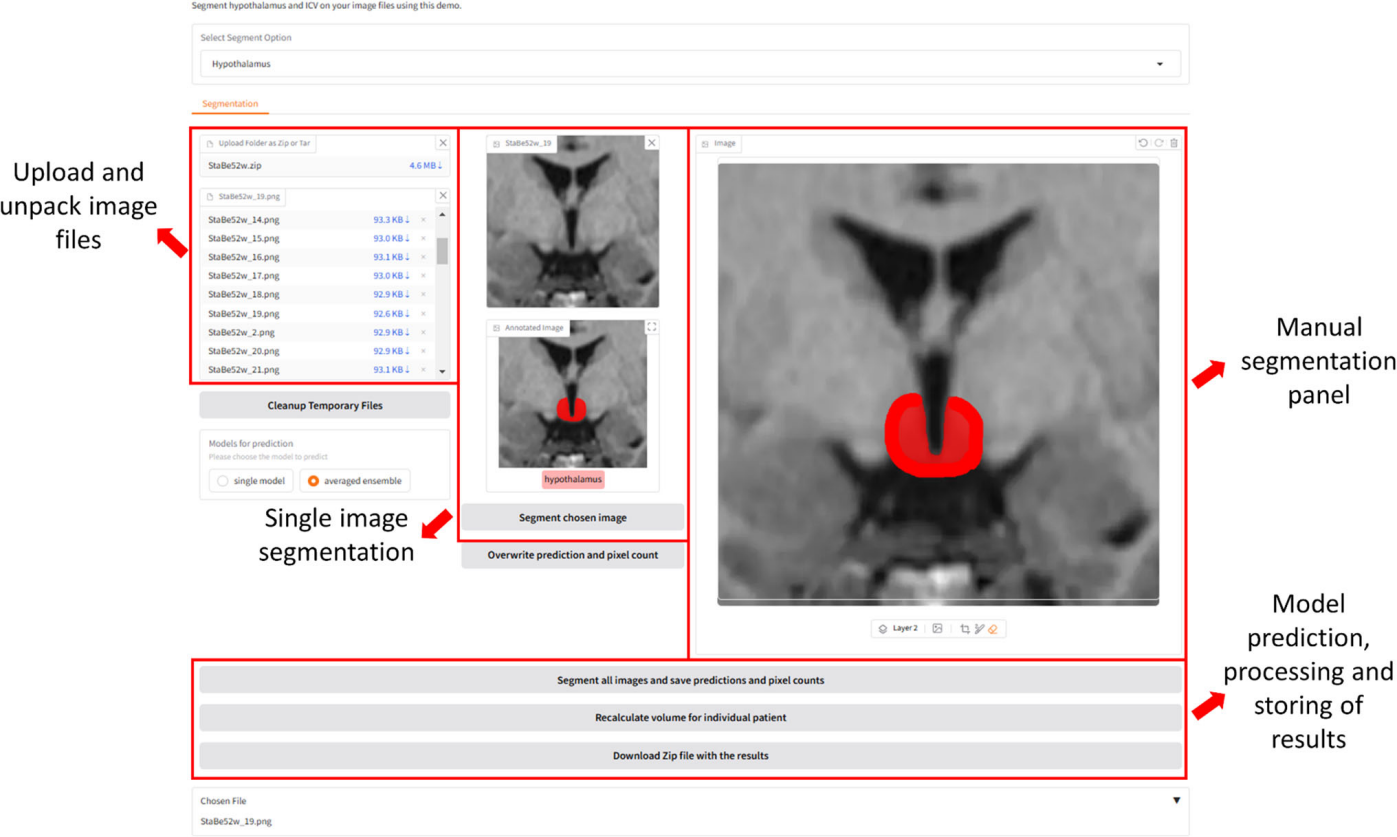
Gradio user interface displaying the hypothalamus segmentation tab

To facilitate the automated and semi-automated segmentation of the hypothalamus and ICV, we have developed a GUI using Gradio^5^ framework, which provides a user-friendly and consistent workflow for both segmentation tasks, incorporating the following key functionalities, which are depicted in Fig. 1:

- *File upload and preprocessing*: Users can upload preprocessed 2D PNG image slices in bulk, which are generated from T1-weighted MRI scans using the *TIFT* software or any other preprocessing software, provided the output matches the input format required by the networks as specified. Upon upload, the files are automatically unpacked, renamed, and sorted according to patient identifiers and slice indices to ensure structured and traceable organization. This preprocessing step minimizes the risk of errors associated with manual file handling and supports scalability for larger datasets.
- *Model selection and automated segmentation*: The interface allows users to choose from a selection of single pre-trained model or an averaged ensemble of five models for segmentation of hypothalamus. The single model is used for prediction of ICV. Then, using the selected model, the GUI performs an automatic segmentation of the hypothalamus or ICV from a batch of input images or a single selected image.
- *Quantitative analysis*: For each segmented image, the system calculates the positive pixel counts, which are then used to estimate the hypothalamic volume or ICV per patient. The results are aggregated and stored in a comprehensive CSV file for downstream analysis. This automated calculation minimizes manual intervention, enhances reproducibility, and provides valuable quantitative insights.
- *Result storage*: The GUI stores individual segmentation masks as grayscale PNG images for each corresponding input image, ensuring traceability and enabling further analysis. The calculated volumes and related metrics are also securely saved in structured formats for integration into broader research workflows.
- *Manual segmentation tool*: Recognizing the need for manual adjustments in cases where automated methods may fall short, the GUI includes an additional segmentation panel equipped with a range of tools for manual refinement. Users can adjust or completely re-segment specific regions to ensure accuracy in challenging cases. Updated segmentation results from the manual adjustment panel can be saved, replacing or complementing the original results. This iterative functionality allows for improved segmentation quality and tailored analysis based on expert input.

**Fig. 2:**
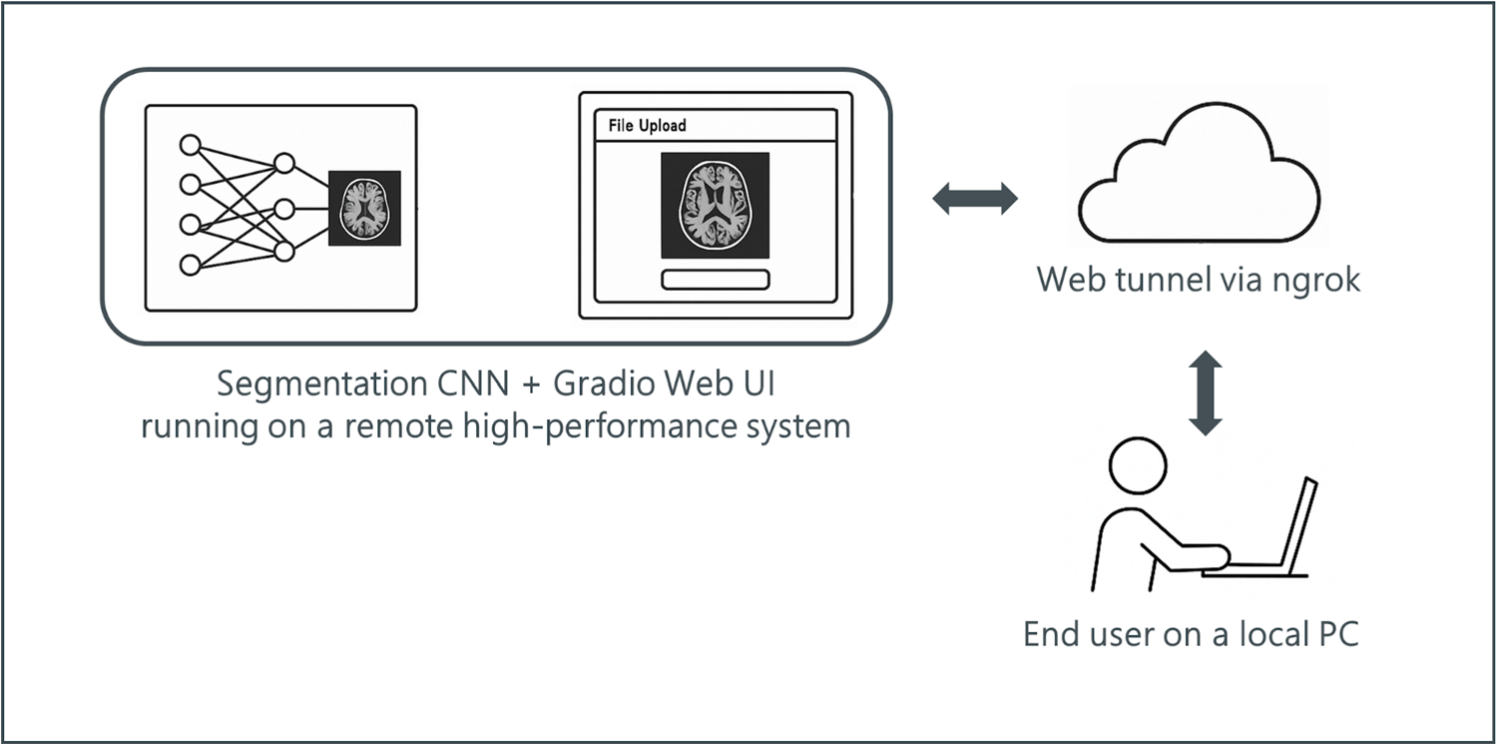
Overview of the remote deployment and access workflow for the CNN-based segmentation tool

The segmentation models are hosted on a high-performance remote system and integrated with a Gradio-based web user interface (UI). External access is enabled through a secure tunnel established using ngrok, which generates a public URL linked to the remote computer. End users can connect to the segmentation tool from any local device via a standard web browser, without the need for software installation or network configuration. This setup ensures platform-independent accessibility, simplifies user interaction, and facilitates seamless collaboration across research teams. The diagram illustrates the complete communication flow from model execution to browser-based interaction (Fig. 2).

### GUI performance evaluation

Evaluation of the GUI was conducted using MRI data from a group of healthy controls (*n* = 39, mean age 68 ± 5 years, 64% male) and patients with Parkinson’s disease (*n* = 90, mean age 69 ± 10 years, 61% male), a neurodegenerative condition known to affect hypothalamic volumes similarly to controls [3]. Segmentation was performed via a shared link, and the quantified hypothalamic volume and ICV were analyzed using descriptive statistics and boxplot visualizations. Additionally, the average prediction time per image was recorded to assess the computational efficiency of the application. In total, 129 MRI volumes from these two groups were included in this pilot case series to evaluate the proposed approach.

## Results

### Performance of hypothalamic segmentation

The results of evaluation of hypothalamus segmentation models are presented in Tables 1 and 2 for ALS test group and healthy control test group, respectively. For the ALS test group, the ensemble model provided consistently strong performance across all evaluation metrics. The segmented volume, Dice score, and volume similarity metrics showed excellent alignment with the ground truth, with the ensemble model achieving the best performance overall. The 95th quantile Hausdorff distance was lower for the ensemble model compared to the individual folds (except for the Fold 2) indicating better boundary accuracy.

**Table 1:** Performance evaluation metrics as average and standard deviation for hypothalamus segmentation in the ALS test group consisting of 15 subjects

Model	Volume [cm^3^]	*p* value (vs. GT)	Dice	HD_95_ [mm]	Volume Similarity
GT (Reference)	0.77 ± 0.09	–	–	–	–
Fold 1	0.79 ± 0.11	0.35	0.83 ± 0.05	2.87 ± 6.68	0.95 ± 0.03
Fold 2	0.80 ± 0.11	0.07	0.84 ± 0.04	**0.90 ± 0.19**	0.96 ± 0.03
Fold 3	**0.76 ± 0.11**	0.67	0.83 ± 0.05	1.07 ± 0.31	0.97 ± 0.04
Fold 4	0.74 ± 0.12	0.24	0.84 ± 0.04	1.09 ± 0.32	0.96 ± 0.05
Fold 5	0.74 ± 0.12	0.18	**0.85 ± 0.03**	1.03 ± 0.22	0.96 ± 0.03
Ensemble	**0.76 ± 0.11**	0.73	**0.85 ± 0.03**	0.93 ± 0.23	**0.97 ± 0.03**

HD_95_: 95th quantile Hausdorff distance; GT: ground truth

Bold values indicate the best-performing model for each metric

**Table 2:** Performance evaluation metrics as average and standard deviation for hypothalamus segmentation in test group of 15 healthy controls

Model	Volume [cm^3^]	*p* value (vs. GT)	Dice	HD_95_ [mm]	Volume Similarity
GT (Reference)	0.86 ± 0.08	–	–	–	–
Fold 1	0.88 ± 0.10	0.39	0.87 ± 0.02	3.96 ± 7.82	0.97 ± 0.02
Fold 2	0.88 ± 0.08	0.19	0.88 ± 0.03	0.84 ± 0.22	**0.98 ± 0.02**
Fold 3	**0.85 ± 0.09**	0.29	0.88 ± 0.03	0.90 ± 0.24	0.97 ± 0.02
Fold 4	0.83 ± 0.08	0.09	0.88 ± 0.02	0.87 ± 0.30	0.96 ± 0.02
Fold 5	0.84 ± 0.10	0.32	0.86 ± 0.04	0.86 ± 0.32	0.96 ± 0.04
Ensemble	**0.85 ± 0.09**	0.29	**0.89 ± 0.02**	**0.80 ± 0.27**	0.97 ± 0.02

HD_95_: 95th quantile Hausdorff distance; GT: ground truth

Bold values indicate the best-performing model for each metric

In the healthy control test group, the ensemble model again demonstrated superior performance compared to the individual folds closely matching the ground truth volume. The Dice similarity coefficient was high for all models, with the ensemble model yielding the highest value, reflecting optimal overlap between the segmented and ground truth volumes. Similarly, the ensemble model achieved the lowest Hausdorff distance, indicating improved precision in delineating the hypothalamus boundaries. Volume similarity also showed strong correlation with the ground truth in the ensemble model.

Overall, the ensemble model outperformed the individual models across measured performance metrics in both the ALS and control groups. Despite relying on z-score normalization for intensity standardization, rather than implying contrast augmentation as in our previous study [15] and thus using a significantly smaller training set for each fold, the performance of the ensemble model in this study was comparable to or even better than the performance of the previously trained single model. Specifically, the Dice coefficient in the current study (0.85 ± 0.03 in ALS, 0.89 ± 0.02 in controls) outperformed the previous work’s model (0.84 ± 0.03 in ALS, 0.86 ± 0.02 in controls). Despite slight increase in HD_95_ values (0.93 ± 0.23 mm in ALS and 0.80 ± 0.27 mm in controls, compared to the earlier study’s 0.82 ± 0.39 mm in ALS and 0.70 ± 0.38 mm in controls), they remain within an acceptable range, indicating that the boundary delineation is still accurate and reliable.

This result suggests that the ensemble approach provides a more reliable and robust segmentation, enhancing consistency and overall accuracy across different training subsets. However, the results also indicate that single model, such as the one from *e.g.* Fold 2, can still achieve adequate segmentation performance in reduced time.

### Performance of ICV segmentation

**Fig. 3:**
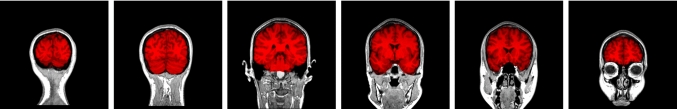
Automatically segmented ICV masks (red) overlaid on preprocessed MRI images in 6 exemplarily chosen slices of single control subject from a test set

The high performance of the ICV segmentation model was evident from the precise delineation of intracranial boundaries, with segmented masks consistently matching the anatomical contours in the MRI scans as visually assessed by overlaying segmented masks on the images (Fig. 3). Quantitative evaluation further confirmed this strong performance. In the test group, the model achieved an average Dice coefficient of 0.92 ± 0.01, a 95% Hausdorff distance of 8.72 ± 2.22 mm, and volume similarity of 0.95 ± 0.03, indicating accurate and consistent segmentations despite moderate inter-subject variability. Predicted ICV volumes (1473.1 ± 41.5 cm^3^) slightly underestimated the ground truth (1618.2 ± 115.8 cm^3^), but remained within a clinically acceptable margin. Based on visual assessment, we have determined that the volume underestimation is primarily attributed to differences between the manual and automated segmentation on edge slices and at the base of the brain, where the boundary of the ICV is not precisely defined and manual delineation applies slightly variable criteria in these inferior regions. However, this underestimation is consistent across all test patients, minimally impacting the overall analysis and ensuring that the segmentation process remains stable.

### GUI Performance in a case series

**Fig. 4:**
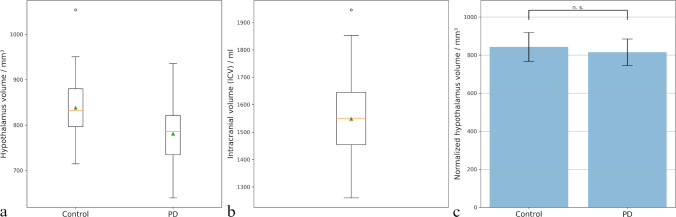
Quantification results in a case series of 39 controls and 90 PD patients. **a** Boxplots of hypothalamus volumes in both groups. **b** Boxplot of ICV across all subjects. Median (orange line) and mean (green triangle) are shown on the box whiskers. **c** Bar plots comparing normalized hypothalamus volume in both groups after manual correction of outliers with black error bars denoting standard deviations and indicated significance calculated using unpaired Student’s *t* test

Figure 4a represents the quantified hypothalamus volume in a pilot study containing data from controls and PD patients. The median volume of the hypothalamus was 832 mm^3^, with an interquartile range (IQR) of 83 mm^3^, indicating a relatively compact distribution of central values. The mean volume was slightly higher at 838 mm^3^, suggesting a slight positive skew. One high-end outlier was identified; however, this outlier is associated with relatively large ICV and disappear after ICV normalization, therefore does not reflect segmentation error or true anomaly.

In the PD group, the median hypothalamic volume was lower than that of controls at 786 mm^3^, with an IQR of 8 mm^3^, mean value of 781 mm^3^ and absence of extreme outliers.

The median ICV across all subjects was 1551 ml, with a mean of 1547 ml, indicating a symmetric distribution of the data. One extreme outlier was observed in the upper range of the distribution. Upon inspection, this case corresponded to a subject with a noticeably larger head size and did not indicate a segmentation error. The relatively large ICV range, as reflected by the whiskers (approximately 679 ml), likely captures the natural biological variability in intracranial volume, which can be attributed to individual differences in brain size, age, sex, and overall population heterogeneity. For the quality control, we identified 5 cases (2 in the control group and 3 in the PD group (including one extreme outlier)), where the automatically segmented ICV deviated by more than 2 standard deviations from the whole group average (separately for males and females) and manually adjusted those cases (manual correction was necessary in 4 cases on total). After manual adjustment median and mean ICV volume in the whole group remained the same (Fig. 4b).

Furthermore, in addition to analyzing the outliers, we performed a systematic visual inspection of approximately 10% of all the analyzed data to further evaluate the accuracy of the segmentations. The automatic segmentation showed a high success rate across both groups. For the hypothalamus, the segmentation achieved a 100% success rate, with no cases requiring manual adjustment. This finding reflects the robustness and reliability of the segmentation model in accurately identifying hypothalamic volumes across all subjects in the study. In the case of ICV, the segmentation tool achieved a success rate of 97%. The remaining 3% of cases exhibited minor under-segmentation at the base of the brain. The dropout rate for the ICV segmentation was therefore minimal, and the tool demonstrated a high level of reliability overall.

After manual correction of ICV volumes, no significantly reduced hypothalamic volume was observed in the PD group compared to the healthy control group (814 ± 70 mm^3^ vs. 842 ± 76 mm^3^, *p* = 0.055) (Fig. 4c), in consistency with previous studies [3].

Average segmentation time per image comprised 354 ms for the hypothalamus utilizing the five-model average ensemble and 87 ms for the ICV utilizing single model for prediction, resulting in a computation time of 18 s per hypothalamus (50 predicted images) and 44 s per ICV (512 predicted images), respectively.

## Discussion

Recent advances in automated imaging segmentation methods, particularly those based on CNNs, have significantly improved the precision and speed of volumetric analyses. However, widespread adoption of CNN-based segmentation models requires intuitive tools accessible to non-experts in machine learning. To address this challenge, we developed an interactive web-based GUI for semi-automatic segmentation of the hypothalamus and ICV using the open-source Gradio library. Through this GUI, researchers can perform segmentation by simply uploading MRI scans, interacting with the interface to make adjustments, and viewing results in near real-time. This tool seamlessly integrates automated and manual workflows, streamlining processes from data upload to volume calculation and result storage, thereby promoting efficiency, reproducibility, and usability in research settings.

Additionally, by integrating ngrok tunneling, the interface can be deployed as a shareable web application, enabling secure, platform-independent access. This functionality facilitates collaboration across institutions and supports reproducibility and scalability in neuroimaging research by eliminating software compatibility issues.

Beyond usability, this study also advances methodology. The current segmentation pipeline introduces z-score intensity normalization and five-fold cross-validation, replacing the contrast-augmented approach and single-validation scheme for training used previously. This new strategy not only standardizes input data but also improves generalization across subjects. Furthermore, instead of using a single model for prediction, we leveraged an ensemble approach that combines the outputs of all five models trained during cross-validation. By aggregating predictions from multiple models, the ensemble helps reduce variance and smooth out individual model biases, leading to more stable and robust segmentation outcomes. This is particularly beneficial when dealing with anatomically small and variable structures like the hypothalamus. As a result, the hypothalamus segmentation demonstrated improved Dice coefficients and maintained a comparable Hausdorff distance (difference to previous study below 1 pixel), indicating both high overlap with ground truth and precise boundary localization.

The tool supports time-sensitive workflows, achieving segmentation speeds of 354 ms per image for the hypothalamus using an ensemble of five models and 87 ms per image for the ICV with a single model, while maintaining robust accuracy.

Segmentation errors identified in the hypothalamic volume analysis, such as occasional outliers, can be addressed via the integrated manual correction tool. Manual adjustments allow researchers to visually inspect and correct automated boundaries, ensuring better alignment with anatomical landmarks and reducing inaccuracies.

This hybrid approach—combining automated segmentation with manual refinement—enhances anatomical accuracy, mitigates segmentation artifacts, and supports reproducibility in studies, especially those involving complex brain structures like the hypothalamus. Although manual corrections are time-intensive, they remain an important component for validating automated results in quantitative research.

Lastly, the inclusion of a proof-of-concept validation case series illustrates the practical utility of the complete workflow in a real-world research context. Taken together, the improved methodology, comprehensive GUI, and applied validation form a meaningful three-fold contribution. This work demonstrates progress toward accessible, robust, and generalizable AI-based neuroimaging tools that meet the needs of both technical and non-technical users alike.

## Conclusions

In summary, the integration of CNN-based hypothalamus segmentation within a Gradio-based GUI holds the potential to democratize access to advanced neuroimaging tools. This approach not only addresses the technical barrier associated with manual segmentation but also enhances the collaborative capacity of the research community. By developing platform-independent, accessible segmentation tools, researchers can more effectively explore and understand the morphology of the hypothalamus.

Furthermore, by designing the tool generically for various applications, it can be adapted and trained for different anatomical structures, extending its utility beyond hypothalamic segmentation. This adaptability fosters broader applications in neuroimaging and other medical imaging domains, empowering researchers to tackle diverse challenges with a single, versatile platform.

## Data Availability

We are open to collaborative projects by reasonable requests to the corresponding author where data can be shared with us for preprocessing based on a data/software sharing agreement. Following this step, access to our developed web-based GUI for automatic segmentation and evaluation can be granted via a shared link.

## Footnotes

1 https://surfer.nmr.mgh.harvard.edu/.

2 https://www.fil.ion.ucl.ac.uk/spm/.

3 https://neuro-jena.github.io/cat//.

4 https://fsl.fmrib.ox.ac.uk/fsl/docs/#/.

5 https://www.gradio.app/.
